# A Gatekeeper Chaperone Complex Directs Translocator Secretion during Type Three Secretion

**DOI:** 10.1371/journal.ppat.1004498

**Published:** 2014-11-06

**Authors:** Tara L. Archuleta, Benjamin W. Spiller

**Affiliations:** 1 Chemical and Physical Biology Program, Vanderbilt University School of Medicine, Nashville, Tennessee, United States of America; 2 Department of Pathology, Microbiology and Immunology, Vanderbilt University School of Medicine, Nashville, Tennessee, United States of America; 3 Department of Pharmacology, Vanderbilt University School of Medicine, Nashville, Tennessee, United States of America; Osaka University, Japan

## Abstract

Many Gram-negative bacteria use Type Three Secretion Systems (T3SS) to deliver effector proteins into host cells. These protein delivery machines are composed of cytosolic components that recognize substrates and generate the force needed for translocation, the secretion conduit, formed by a needle complex and associated membrane spanning basal body, and translocators that form the pore in the target cell. A defined order of secretion in which needle component proteins are secreted first, followed by translocators, and finally effectors, is necessary for this system to be effective. While the secreted effectors vary significantly between organisms, the ∼20 individual protein components that form the T3SS are conserved in many pathogenic bacteria. One such conserved protein, referred to as either a plug or gatekeeper, is necessary to prevent unregulated effector release and to allow efficient translocator secretion. The mechanism by which translocator secretion is promoted while effector release is inhibited by gatekeepers is unknown. We present the structure of the *Chlamydial* gatekeeper, CopN, bound to a translocator-specific chaperone. The structure identifies a previously unknown interface between gatekeepers and translocator chaperones and reveals that in the gatekeeper-chaperone complex the canonical translocator-binding groove is free to bind translocators. Structure-based mutagenesis of the homologous complex in *Shigella* reveals that the gatekeeper-chaperone-translocator complex is essential for translocator secretion and for the ordered secretion of translocators prior to effectors.

## Author Summary

Type Three Secretion Systems (T3SS) are essential virulence factors found in many pathogenic Gram-negative bacteria. These machines aid infection by delivering bacterial proteins into host cells where these proteins modulate host processes and help establish a niche for the bacteria. Protein delivery occurs in a highly regulated manner in which proteins involved in early steps in infection, or necessary to build the secretion conduit, are typically secreted before other substrates, a phenomenon termed secretion hierarchy. This study presents the structure of a molecular complex that physically links one class of early substrates, components of the secretion pore termed translocators, to a gatekeeper protein, a protein that has been implicated in the secretion hierarchy. Disruption of this interaction in *Shigella* disrupts the secretion of translocators, while supporting increased secretion of effectors, resulting in phenotypes indistinguishable from a gatekeeper deletion, and leading to the conclusion that a gatekeeper-chaperone-translocator complex is a critical component of the T3SS.

## Introduction

Type Three Secretion Systems (T3SS) are conserved bacterial protein delivery machines used by many pathogenic Gram-negative bacteria to deliver a diverse group of protein molecules, termed effectors, into cells [1]–[4]. The type three secretion (T3S) apparatus is a conserved molecular machine that forms a protein-conducting channel from the bacterial cytosol to the target cell cytosol. Major structural components of the T3SS include: a cytosolic-ring complex, which includes the ATPase that catalyzes protein unfolding and secretion; a basal-body, which forms a pore across both the inner and outer bacterial membranes; a needle complex, which extends from the basal-body to the host cell; and translocators, which form a pore in the target cell membrane, termed a translocon [5], [6].

The translocon, through which effectors enter the host cell, is an oligomeric pore formed by bacterial proteins termed translocators that are themselves secretion substrates of the T3SS [2]. Translocator secretion is regulated such that it occurs prior to effector secretion, ensuring that effector secretion occurs after a functional conduit from the bacterial cytoplasm to the target cell has been formed [7], [8]. Efficient secretion is dependent on the interaction of specialized chaperones with cytosolic T3SS components [9]. Molecular structures have revealed two architectures for T3S chaperones: a mixed α/β homo or heterodimer and an all α-helical tetratricopeptide repeat (TPR) chaperone [10]–[14]. These chaperones, termed class I (α/β) and class II (TPR), are specific for effectors and translocators, respectively [9]. Our understanding of how the T3SS switches from translocator to effector secretion is limited, but in multiple systems this process is known to involve a conserved gatekeeper protein [15]–[20]. Gatekeepers are encoded as one of two molecular architectures, either as two separate proteins (YopN-TyeA family), or as a gene fusion (MxiC family) [20]. The importance of this architectural distinction is unknown, and a protein fusion resulting from ribosomal frameshifting has been reported without an evident functional change [21].

In *Chlamydia*, the gatekeeper, CopN, is known to directly bind Scc3, a translocator-specific chaperone [22]–[26]. Translocator-specific chaperones (class II chaperones) bind directly to translocators, prevent their degradation, and maintain the translocators in a secretion competent state [9], [13]. Structures from homologous class II chaperone/translocator pairs have revealed the chaperone to be a TPR protein with a conserved binding groove that binds an amino terminal chaperone-binding motif on the translocator [12]–[14], [27]. In addition to binding translocators, Scc3 also binds CopN, although the nature of this interaction is unknown [22], [23], [25], [26]. It is not known if CopN and the *Chlamydial* translocators (CopB/B2) compete for the TPR binding groove on Scc3, or if different binding determinants are responsible for the Scc3-CopN interaction. More fundamentally, it is not known how gatekeepers promote translocator secretion.

Gatekeepers and translocator chaperones have been observed in immunopurified complexes from other systems, but only as components of large complexes that also include other components of the T3SS [15], [17], [28]. Because such complexes are not readily accessible to structural study, we have focused our structural studies on the gatekeeper-translocator chaperone complex from *Chlamydia*. We reasoned that the CopN-Scc3 complex is likely to be involved in the ordered secretion of translocators prior to effectors, a conserved phenomenon termed the translocator-effector secretion hierarchy.

The origin of the translocator-effector secretion hierarchy is not understood, but has been proposed to arise from differential affinities and competition for binding sites either between chaperones and their effector or translocator cargo or between chaperone-effector/translocator complexes and cytosolic components of the T3SS [10], [11], [29]–[32]. To assess the importance of gatekeeper-translocator chaperone interactions in diverse pathogens, and because adequate tools and reagents for functional analysis of CopN mutants are not available in *Chlamydia*, we have extended our structural analysis with functional studies of MxiC and IpgC, the gatekeeper and translocator-specific chaperone from *Shigella*.

## Results

### Structure of Scc3-CopN complex

**Figure 1:**
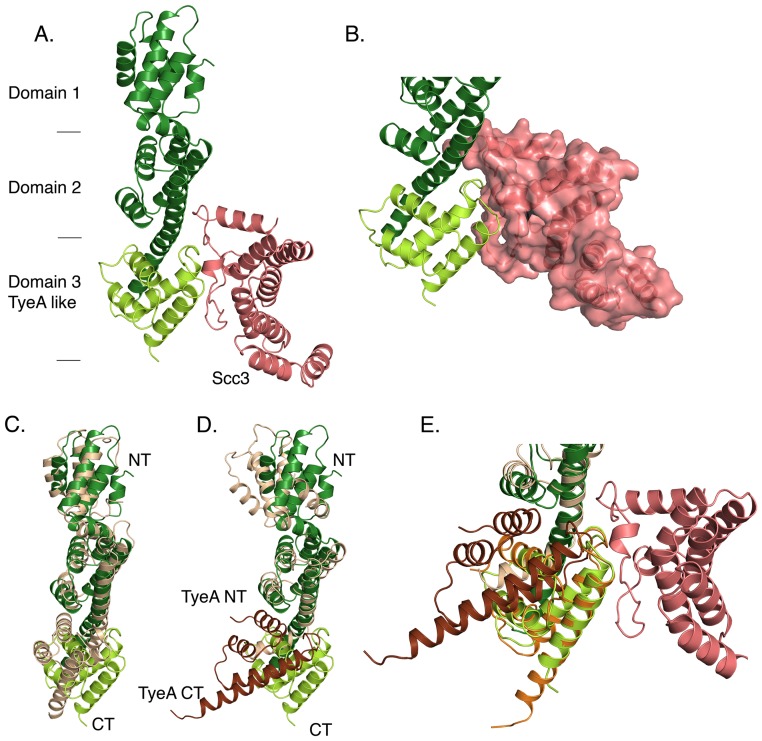
Crystal structure of Scc3-CopN_Δ84_. CopN is colored green, with the YopN homology region in dark green and the TyeA homology region in light green. **A.** A ribbon diagram of the Scc3-CopN_Δ84_ structure. Approximate domain boundaries are indicated. Scc3, salmon, binds across the domain 2-domain 3 domain interface. **B.** A close-up of the Scc3-CopN_Δ84_ interface, oriented as in **A**, with Scc3 shown as a molecular surface. Scc3 forms a relatively flat surface that bridges domains 2 and 3 of CopN_Δ84._ **C., D.** Comparisons of CopN and homologs. **C.** Comparison between MxiC and CopN. MxiC is colored tan and shown with thin helices. **D.** Comparison between CopN and the YopN/TyeA complex. YopN is tan and TyeA is brown. YopN and TyeA are and shown with thin helices. **E.** Overlay of TyeA in orientation shown in **D.** and when aligned as a rigid body to the carboxy terminal 91 residues of CopN (rmsd = 0.4 Å).

We determined the crystal structure of the Scc3-CopN_Δ84_ complex and refined the structure to 2.2 Å (PDB ID 4NRH). The amino terminal 84 residues of CopN were not included in the construct used for crystallization because they are unstructured [25]. Data collection and structure refinement statistics are given in Table 1, and representative electron density is shown in Supporting Figure S1. Two nearly identical Scc3-CopN_Δ84_ complexes (RMS deviation 0.34 Å for all CopN_Δ84_ mainchain atoms and 0.49 Å for all Scc3 mainchain atoms) are present in the asymmetric unit. CopN_Δ84_ forms a long cylindrical structure composed of three helical domains (Figure 1). A search for structurally similar proteins using the DALI software [33], indicates structural homology to the globin fold, which, aside from the use described here, is used in bacteria both as a light harvesting complex and as a stress response sigma factor [34]–[36]. In gatekeeper proteins multiple domains are concatenated through elongated connecting helices, whereas globin domains typically oligomerize through lateral contacts. CopN_Δ84_ is structurally similar to other gatekeeper proteins, both MxiC from *Shigella* and the YopN-TyeA complex from *Yersinia*. The most substantial differences among family members relate to the position of the carboxy-terminal domain or subunit (Figure 1 and [37], [38]). In the Scc3-CopN_Δ84_ complex, this domain is translated ∼9.5 Å and rotated ∼50° relative to the YopN-TyeA complex (Figure 1). Similarly, Scc3 is structurally similar to other translocator chaperones. The striking result from the Scc3-CopN_Δ84_ is the unexpected assembly of the complex and the role of the Scc3 amino terminus in binding CopN_Δ84_.

**Table 1:** Data collection, phasing, and refinement statistics.

	Au(CN)_2_	Native (2.4 Å data)	Native (all data)
**Data collection**			
Space group	P2_1_	P2_1_	P2_1_
Cell dimensions			
*a*, *b*, *c* (Å)	62.88,	61.57,	61.57,
	97.06,	96.18,	96.18,
	94.36	94.47	94.47
α, β, γ (°)	90.0,	90.0,	90.0,
	91.87,	92.27,	92.27,
	90.0	90.0	90.0
Wavelength	1.00798	0.97872	0.97872
Resolution (Å)^a^	2.8 (2.85–2.80)	2.4 (2.49–2.40)	2.2 (2.28–2.2)
*R*_sym_ a ^,^ b	8.8% (55%)	5.7 (34.0)	6.2 (55.4)
*I/*σ*I* a	22.5 (3.25)	28.4 (4.46)	21.1 (2.4)
Completeness (%)^a^	100 (99.7)	98.0 (82.2)	89.7 (55.4)
Redundancy^a^	10.9 (9.6)	7.7(6.3)	7.3 (4.5)
**Refinement**			
Resolution(Å)^a^			50-2.2 (2.25-2.2)
No. reflections^a^			50,339 (1,807)
*R*_work_/*R*_free_ c ^,^ d			18.5/22.2
No. atoms			
Protein			7,302
Ligand/ion			2 Na^+^
Water			414
*B*-factors (Å^2^)			
Protein			49.8
Ligand/ion			51.1
Water			48.6
R.m.s deviations			
Bond lengths (Å)			0.006
Bond angles (°)			0.9
Ramachandran			
Outliers			0%
Allowed			1.21%
Favored			98.79%

a Parenthesis indicate outer-shell statistics.

b *R*_sym_ = ∑*hkl*∑*i*|*Ihkl,i*−<*Ihkl*>|/∑*hk∑i Ihkl,i Ihkl,i* is the scaled intensity of the ith measurement of reflection *h*, *k*, *l*,.

<*Ihkl*> is the average intensity for reflection *I*, and *N* is the number of observations of *I*.

c *R*_cryst_ = ∑*hkl*|*F_o_*−*F_c_*|/∑*hkl*|*F_o_*|, where *F_o_* and *F_c_* are the observed and calculated structure factors.

d *R*_free_ was calculated as for *R*cryst, but using 5% of data, randomly chosen and excluded from refinement.

**Figure 2:**
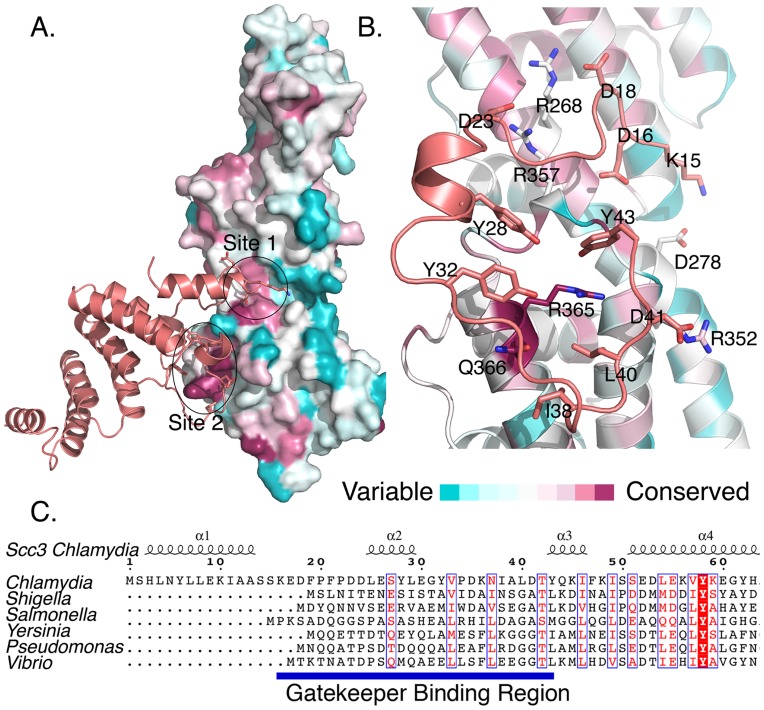
Sequence conservation in CopN and Scc3. **A., B.** Sequence conservation displayed on the CopN_Δ84_ structure. Residues are colored by conservation (pink is conserved, blue is variable). **A.** Scc3 interacts with two conserved regions, site 1 and site 2, on CopN. **B.** An expanded view of the CopN-Scc3 interface. For clarity only the GBR region is shown. The interface is primarily composed of hydrophobic residues from Scc3 that surround a conserved arginine (R365) on CopN. **C.** Sequence conservation within GBR's is minimal. Scc3 homologs from multiple species are aligned based on conservation throughout their sequences, revealing that the GBR region is present, but not highly conserved in homologs. Sequences used are from *C. pneumonia, S flexneri, S. enterica, B. pertussis, Y. enterocolitica, P. aeruginosa, V. parahaemolyticus*. Multiple sequences from each genera used in **C.** were used in **A.** and **B.** (see methods).

T3SS chaperones bind the amino terminus of effectors and translocators, and class II chaperones (those specific for translocators) use a conserved peptide-in-groove binding mode, utilizing the TPR binding groove, in which translocators bind in the concave face of the chaperone [9]–[14], [27]. The structure reveals that Scc3 does not engage CopN_Δ84_ using this conserved binding groove. Instead, the amino terminus, referred to here as a gatekeeper-binding region (GBR), forms a relatively flat surface, adjacent to the convex side of the TPR and binds across the interdomain interface of the last two domains of CopN_Δ84_ (Figure 1). The interface formed by this interaction results from burial of 980 Å^2^ of surface area. The Scc3 side of the interface is formed exclusively by residues from the GBR, consistent with separate functions of translocator and gatekeeper binding for the TPR and GBR regions of Scc3. Despite minimal sequence conservation, other translocator chaperones also have an amino terminal extension (GBR) prior to the TPR domain (Figure 2). In the homologs from *Shigella* and *Pseudomonas* this region mediates homo-dimerization, although translocator binding is known to disrupt these dimers such that in translocator-chaperone complexes this region (GBR) is no longer involved in homodimerization [13], [29], [39]. In the homologs from *Yersinia* and *Pseudomonas* crystallization and structure determination required removal of the GBR [12], [14], [27].

### The Scc3-CopN interface is conserved in other bacteria

Scc3 engages CopN_Δ84_ with residues from the GBR, which bind a contiguous surface on CopN_Δ84_. This surface is formed by two distant patches of sequence conservation, site 1 and site 2, and spans the second and third domains of CopN_Δ84_, requiring these two domains to be appropriately oriented (Figures 1, 2). In the YopN-TyeA family, the third domain is encoded as a separate protein, such that in these homologs one would predict the interdomain interface recognized by the Scc3 homolog to be a dimer.

Residues 16–23 of Scc3 interact principally with site 1 of CopN_Δ84_, whereas residues 24–43 form a much larger interface in which Scc3 projects a ring of hydrophobic sidechains toward CopN_Δ84_ to surround a highly conserved arginine (R365) on CopN_Δ84_ (Figures 2, Supporting Figure S2). This interaction includes three tyrosines, one of which, Y43 is oriented to allow a π-cation interaction. Peripheral to this ring of hydrophobic residues are a collection of inter-molecular salt bridges (Figure 2). The circumscribed arginine (R365) is conserved across diverse species, including species with two polypeptide gatekeepers and is among the most conserved surface exposed residues in this protein family (this residue is an arginine in homologs from *Shigella*, *Vibrio*, *Pseudomonas*, *Bordetella*, and *Yersinia* and glutamine in *Salmonella*) (Supporting Figure S2). Residues on the CopN side of this interface are better conserved than those on the GBR, despite the fact that they span two proteins in the *Yersinia* architecture and are on the same protein in the architecture presented here (Figure 2, Supporting Figure S2).

To assess the importance of the two binding regions, we disrupted each interaction by mutagenesis. We made an amino terminal 24 amino acid deletion to Scc3 (Scc3_Δ24_), which eliminates the GBR-site one interaction. We also mutated the central arginine and two adjacent residues (A362R, R365D, and G369R) in site 2 of CopN_Δ84_ (CopN_Δ84_-RDR). A362, R365, and G369 are buried by Scc3 and likely solvent exposed in unliganded CopN_Δ84_. We introduced charged residues at these sites with the expectation that the solvent exposed charges would not disturb CopN, but would disrupt the CopN_Δ84_-Scc3 complex. CopN_Δ84_-RDR and Scc3_Δ24_, are well-folded, as judged by circular dichroism spectra similar to CopN_Δ84_ and Scc3 (Supporting Figure S3, Methods S1). As judged by the inability of Scc3_Δ24_ to bind CopN_Δ84_ and the inability of CopN_Δ84_-RDR to bind Scc3 (Supporting Figure S4), both regions are important for Scc3-CopN_Δ84_ complex formation.

### The translocator-binding site in Scc3 is available in the Scc3-CopN complex

**Figure 3:**
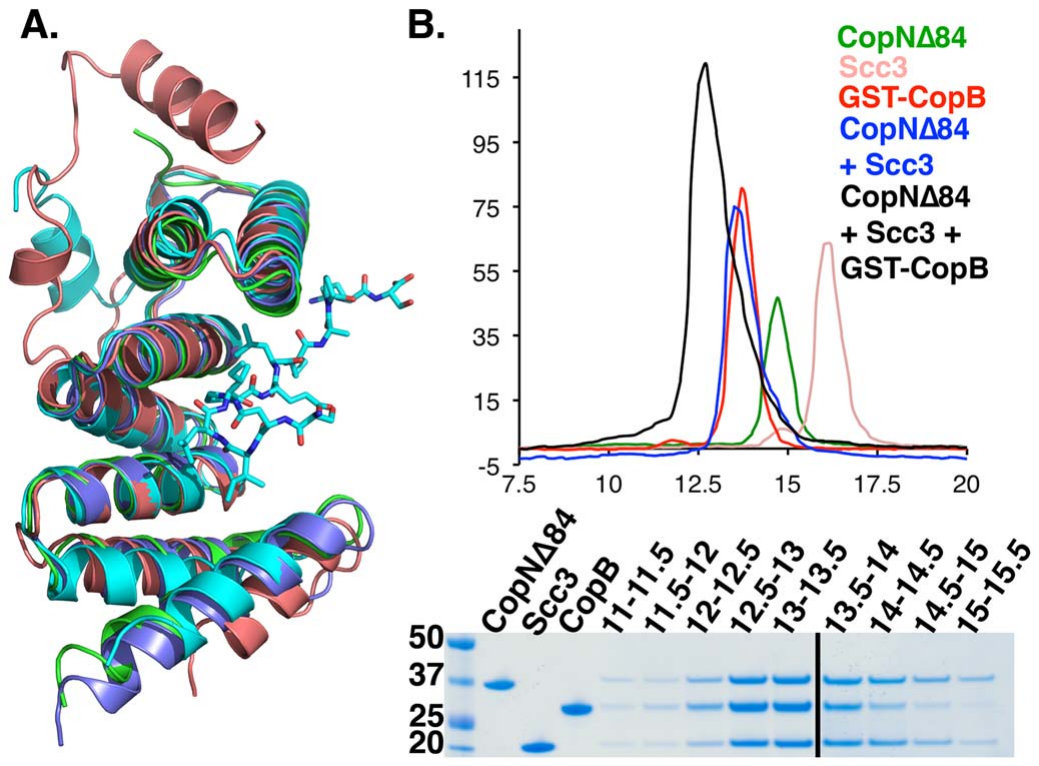
The Scc3-CopN_Δ84_ complex binds directly to translocators. **A.** An overlay of class II T3S chaperones. All structures except the Scc3 structure were determined bound to translocator peptides. For clarity, only the IpaB peptide from *Shigella* is shown. Scc3 is shown in salmon, IpgC (*Shigella*) in teal, PcrH (*Pseudomonas*) in green, and SycD (*Yersinia*) in blue. The peptide-binding site is conserved and open in Scc3. **B.** CopN-Scc3 complex directly binds to the CopB translocator. Top: gel filtration traces reveal that the ternary complex is tight enough to survive gel filtration. Bottom: SDS PAGE confirming complex formation.

TPR family proteins are often unfolded when not bound to appropriate ligands and are considered to be somewhat flexible proteins [40], [41]. Scc3 has an appropriately organized but empty binding cleft when bound to CopN. Structural comparison with other class II chaperones, for which structures have been determined in complex with translocator-derived peptides, indicates that CopN_Δ84_ binding causes no significant reorganization of the translocator-binding site (Figure 3A). The Scc3-CopN_Δ84_ binding mode leaves the translocator-binding site on Scc3 unperturbed and available to bind translocators (Figure 3A). In support of this observation, the purified Scc3-CopN_Δ84_ complex is able to directly bind a translocator-derived peptide (presented as residues 158–177 from CopB fused to GST) and form a CopN_Δ84_-Scc3-CopB_158-177_ complex as judged by size exclusion chromatography (Figure 3B). Isothermal Titration Calorimetry (ITC) using a synthetic peptide (CopB residues 163–173) revealed Kd's of 79±16 µM for the Scc3-CopB peptide complex and 49±13 µM for the Scc3-CopN_Δ84_ peptide complex (Supporting Figure S5, Methods S1).

### Disruption of the Scc3-CopN interface alters secretion in *Shigella*

**Figure 4:**
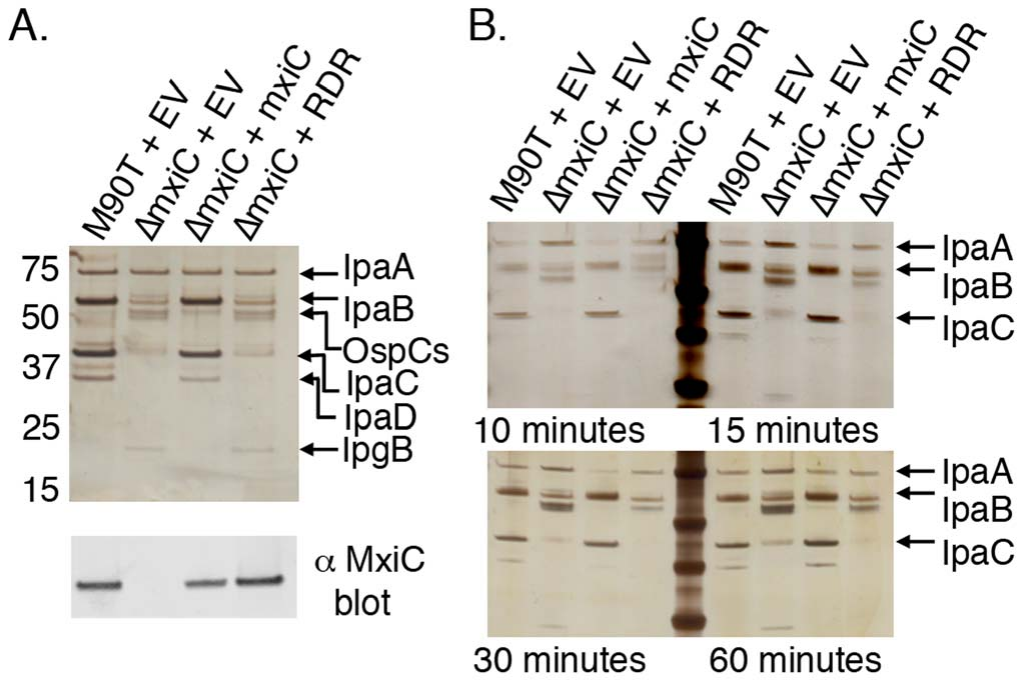
The gatekeeper-chaperone complex is needed for efficient translocator secretion. **A.** T3S was induced from wild type and mxiC mutant *Shigella* strains. M90T is a wild type strain. ΔmxiC is M90T derived, with mxiC deleted [18]. EV: empty vector. Strains were complemeted with mxiC, mxiC mutant (RDR), or empty vector (EV). Proteins were visualized by silver staining and identified by MS. IpaA, OspCs, and IpgB are effectors. IpaB, IpaC, and IpaD are translocators. Bottom: anti-MxiC blot of the same samples indicates that MxiC and MxiC-RDR are secreted normally. **B.** A secretion timecourse. The experiment shown in **A.** was repeated and samples taken a 10, 15, 30, and 60 minutes post induction. IpaA, an effector, is secreted at early time points in the absence of functional Mxic, but in the presence of MxiC it is not efficiently secreted until later time points.

To determine the importance of the gatekeeper-chaperone interaction during T3S, we disrupted the homologous gatekeeper-translocator chaperone interface in *Shigella*. *Shigella*, unlike *Chlamydia*, are genetically tractable allowing disruption of the endogenous mxiC gene (the copN homolog) and rescue with a plasmid expressing mutant or wild-type MxiC. This is a well-established strategy that has been used to study other MxiC mutants [18], [28]. The CopN and Scc3 homologs in *Shigella*, MxiC and IpgC, form a complex that includes the T3SS ATPase [28]. The Scc3-CopN_Δ84_ structure was disrupted by mutation of A362R, R365D, and G369R on CopN (Supporting Figure S4), supporting the idea that the homologous mutations would disrupt IpgC-MxiC interface. We expressed the E331R/R334D/I338R MxiC mutant (MxiC-RDR) from a plasmid in a previously described mxiC null *Shigella* strain [18] and compared secretion profiles following Congo Red induction. MxiC-RDR is deficient in secretion of the translocators IpaB, IpaC, and IpaD, but efficiently secretes IpaA, an effector, and secretes elevated levels of the effectors OspC1-3 and IpgB (Figure 4A). IpaA is not secreted efficiently if wild type MxiC is present, but is secreted earlier and in greater quantities from ΔMxiC or MxiC-RDR strains (Figure 4B, compare IpaA secretion at 10 minutes and 60 minutes). The secretion profile of MxiC-RDR closely mimics that of the ΔMxiC strain (Figure 4A), highlighting the importance of gatekeeper-translocator chaperone complexes in translocator secretion. To further evaluate the importance of the conserved, central, arginine, we evaluated the secretion profile of a single point mutant, MxiC-R334D, revealing a phenotype similar to the triple mutant (Supporting Figure S6). Similar to CopN, MxiC is both the gatekeeper and a T3S substrate [18], [42], [43]. To verify that the mutations to MxiC did not prevent recognition and secretion of MxiC by the T3SS, we evaluated the secretion of MxiC-RDR, which is unaltered from wild-type MxiC (Figure 4A). To further confirm that the mutations didn't grossly alter the structure of MxiC, we compared circular dichroism specta of MxiC_Δ74_, MxiC_Δ74_-RDR, and MxiC_Δ74_-R334D, which indicated that all three proteins are similarly folded (Supporting Figure S3, Methods S1). In these constructs the first 74 residues of MxiC have been deleted to allow expression and purification from *E. coli*. MxiC-RDR is secreted in a similar manner to MxiC, indicating that the mutations do not disrupt its ability to interact with the T3SS, yet is unable to direct translocators for secretion, and is unable to prevent inappropriate secretion of the effectors, IpaA, OspC1-3, and IpgB.

## Discussion

In this study we have presented the structure of CopN_Δ84_ bound to Scc3. This structure provides the first description of a novel interaction for translocator-chaperones, which involves an amino-terminal extension, termed the GBR, binding across the YopN-TyeA-like domain interface in CopN_Δ84_. The translocator-chaperone-gatekeeper interaction involves a conserved arginine from the gatekeeper and the amino terminal GBR from the chaperone. Despite the lack of sequence conservation within the chaperone, the presence of a GBR in class II chaperones appears to be conserved (Figure 2). Scc3's GBR is ∼20 amino acids longer than others, implying that the extensive interactions made to the YopN-like domain are likely unique to the Scc3-CopN_Δ84_ complex. Although the unique α1 helix of Scc3 (amino acids 2–15) does not contact CopN, α1 does orient the α1–α2 loop, which contacts CopN. In organisms with shorter GBRs, other T3S apparatus components likely contribute to organizing this interface. This appears to be the case in *Salmonella*, where the gatekeeper interacts with the translocator-chaperone when it is bound to either translocator, but not to the empty chaperone [17]. In *Shigella* and *Pseudomonas*, the translocator-chaperones are known to homodimerize using the amino terminus, and translocator binding is necessary to disrupt this dimer [29], [39]. Although the monomeric chaperone is able to bind translocators, our data suggest a role for the chaperone amino terminus distinct from the role in homodimerization, namely a role in gatekeeper binding. We find that Scc3 is a monomer and the Scc3-CopN_Δ84_ complex is a heterodimer in solution (Supporting Figure S7). We suggest that disruption of chaperone homodimers, by translocator binding likely occurs prior to chaperone-gatekeeper complex formation, and that once the chaperone-translocator complex is formed, it is recruited to the T3S apparatus by the gatekeeper.

The binding mode observed in our structure leaves the canonical translocator-binding groove free and available to bind translocators. The Scc3-CopN_Δ84_ complex binds directly to CopB (Figure 3, Supporting Figure S5), a *Chlamydial* translocator, thus establishing a physical link between the gatekeeper and a translocator. By physically linking gatekeepers and translocators, the temporal order of secretion events in which translocators are secreted immediately after gatekeepers and prior to effectors is assured. The mechanism by which gatekeepers are recruited to the apparatus prior to effectors or translocators remains to be determined. The unusual CopN-Scc3 interface and relatively small buried-surface area (980 Å^2^) are consistent with the physiological role of this complex, in that the gatekeeper-translocator chaperone must assemble and disassemble each cycle of translocator secretion.

In *Chlamydia* the CopN-Scc3 interaction can be formed with purified proteins, suggesting that it is stronger than in homologous systems, likely because the GBR is longer and complex formation is not prevented by chaperone homodimerization. The precise advantage this affords *Chlamydia* is unclear, but T3S activation may represent an early “committed step” for *Chlamydia* infection. As obligate intracellular pathogens, the need for *Chlamydia* to enter their host subsequent to T3S activation seems absolute. *Chlamydia* are dependent on their hosts for ATP [44] and therefore a T3S event that doesn't result in entry is likely fatal for the bacterium. Consistent with this idea, *Chlamydia* also express a second translocator chaperone, Scc2, which is expressed during late stages of infection, after invasion, and does not bind CopN [22], [23].

Mutations shown to disrupt the CopN-Scc3 complex were evaluated in the MxiC-IpgC complex. The highly conserved central arginine and two additional charged residues, located at positions homologous to the sites mutated in CopN, were mutated (E331R/R334D/I338R) on a MxiC expressing plasmid and used to complement a mxiC deletion strain. This mutant MxiC (MxiC-RDR) was expressed and secreted normally, indicating that the mutations did not significantly disrupt MxiC. Strains harboring MxiC-RDR, however, mimicked deletion strains and were both deficient in translocator secretion and secreted elevated levels of effectors, resulting in significant effector secretion at early timepoints, prior to translocator secretion. These results support the conclusion that a key function of MxiC during secretion is to scaffold translocators and that the MxiC-IpgC complex is needed for this function. This arrangement, in which the gatekeeper directs translocators to the secretion apparatus, makes proper assembly of a gatekeeper-chaperone-translocator complex needed to promote translocator secretion. Gatekeepers are also needed to prevent effector secretion [15]–[20] indicating that the relevant “plug” that prevents premature effector secretion is likely the gatekeeper-chaperone-translocator complex. Consistent with this interpretation, disruption of IpaB (a *Shigella* translocator) or MxiC (the gatekeeper) both cause constitutive effector secretion [18]. In many systems, translocator chaperones have an additional role and act as transcription co-activators after translocator secretion thereby linking expression of some effector proteins to secretion of translocators [9].

Collectively, our results support a new mechanism for the translocator-effector hierarchy. We suggest that the translocators are recruited to the T3S pore as a molecular complex including the gatekeeper, translocator-chaperone, and translocator. There are likely multiple variations on this theme, one of which is evident in *Pseudomonas aeruginosa*, where translocators make multiple interactions with their chaperone, including regions outside the canonical chaperone binding region, at the extreme carboxy terminus [45]. The entire complex is needed both to promote translocator secretion and to prevent effector secretion. A triggering event from the tip of the T3S needle is known to induce gatekeeper release [18], which through the molecular complex described above is directly linked to translocator secretion. Secretion of the gatekeeper and translocator then allows effector secretion through a gatekeeper independent mechanism, similar to the efficient effector secretion seen in gatekeeper mutants.

## Methods

### Purification of His tagged proteins

CopN_Δ84_ and Scc3, from *C. pneumoniae* AR39, and MxiC_Δ74_ from *S. flexneri* were amplified and cloned into pET28 with an amino-terminal hexa-histadine tag. CopN_Δ84_ and MxiC_Δ74_ are amino terminal 84 and 74 amino acid deletions of CopN and MxiC, respectively. It was previously found that these were well-behaved variants [25], [37]. All CopN variants were made in pET28 using PCR based mutagenesis and verified by sequencing. CopN truncation mutants were designed from described limited proteolysis mass spectrometry analysis [25]. Proteins were expressed in BL21 *(DE3)* bacteria grown in Luria Broth at 37°C. Bacteria at an optical density (at 600 nm) of ∼0.6 were induced at 20°C with 0.1 mM isopropyl β-D-thiogalactopyranoside, and grown for ∼12 hours. Cultures were harvested by centrifugation and lysed with a French Press in phosphate buffered saline with ∼1 µg/mL chicken egg white lysozyme, ∼1 µg/mL bovine pancreatic deoxyribonuclease I, 10 µg/mL leupeptin, 1 mM PMSF, 0.7 µg/mL pepstatin. The lysate was clarified by centrifugation and proteins were purified by Co-NTA affinity using Talon resin. Eluted proteins were further purified by size exclusion chromatography, superdex 200, before being snap-frozen in liquid nitrogen and stored at −80°C until needed.

### Expression and purification of GST-CopB

The Scc3 binding region of CopB, residues 158–178 based on sequence homology with IpaB from *Shigella* [13], was expressed as a GST fusion protein from PGEX-4T. Proteins were expressed and purified as for His tagged proteins with minor modifications; proteins were expressed from BL21 bacteria and purified with Glutathione Sepharose 4B (GE Healthcare).

### Gel-filtration assays

Chaperone, and translocator peptide binding assays were performed by gel-filtration, using a 24 mL Superdex 200 10/300 GL (GE Healthcare), equilibrated in 10 mM Tris-HCl pH 7.5, 150 mM NaCl, run at 0.5 mL/min, and maintained at 4°C. Equivalent molar concentrations, determined from calculated extinction coefficients, of proteins were applied to the gel filtration column. Protein complexes were incubated for 15 minutes prior to analysis. Molecular weight determination was done under the same conditions using gel-filtration standards from BioRad.

### Crystallization and preparation of heavy atom derivatives

Scc3-CopN_Δ84_ crystals were grown via vapor diffusion from a reservoir containing 0.2 M Na/K tartrate and 18–22% PEG 3350. Crystals were obtained from a 1∶1 mixture of reservoir and 15 mg/MmL Scc3-CopN_Δ84_. KAu(CN)_2_ derivatives were prepared by adding 100 mM KAu(CN)_2_ to the crystal drop to a final concentration of 2 mM KAu(CN)_2_. After two days of incubation derivative crystals were harvested. Native and derivative crystals were cryoprotected with 15% glycerol and flash cooled.

### Data collection, structure determination, and analysis

Diffraction data were collected from single crystals on stations D and F at LS-CAT beam line at the Advanced Photon Source. Data were indexed, integrated and scaled with HKL2000 [46]. Three gold atoms were located and refined using Phenix [47]. The initial figure of merit for these sites was 0.46, which improved to 0.70 following density modification. The model was traced with a combination of automated and manual building in Phenix and COOT [47], [48]. Multiple rounds of refinement were done using Phenix. Refinement included simulated annealing, coordinate, individual B-factor, and TLS refinement as implemented in Phenix [47]. Non-crystallographic symmetry constraints were included in all rounds of positional refinement. Data collection and refinement statistics are given in Table S1. Figures were prepared using Pymol, ClustalW, ESPript, the DALI server, the PISA server, and the Consurf server [33], [49]–[52].

### Alignments

Sequences used in the Consurf alignments of CopN homologs were chosen to represent the sequence diversity within genera shown in Figure 1 and included five *Chlamydial* sequences (NP_224529.1, NP_829326.1, YP_515466.1, 84785886, 332806765, YP_005809291.1), four *Shigella* sequences (YP_005712038, YP_313363.1, YP_001883209.1, YP_406185.1), three *Salmonella* sequences (1236849, 75349427, NP_461818.1), and three *Bordetella* sequences (NP_880900.1, WP_004568105.1, NP_884470.1). For the two-component gatekeepers, chimeric sequences were generated to agree with the spatial orientations in the YopN/TyeA structure (pdb accession code 1XL3). *Yersinia* YopN sequences used were NP_863522.1, NP_395173.1, and NP_052400.1. Because there is zero sequence diversity in TyeA from species evaluated here, we used YP_004210060.1 for all chimeras. An identical strategy was used for *Pseudomonas* and *Vibrio*. *Pseudomonas* sequences used were NP_250389.1, WP_003122865.1, and WP_010794024.1, WP_015648550.1, which were all matched with WP_009876220.1. *Vibrio* sequences used were NP_798046.1, YP_003285992.1, WP_005395115.1, WP_005377238.1, WP_005441804.1, WP_004745560.1, WP_005528936.1, which were all matched with WP_005395113.1.

### *Shigella* secretion assay

Secretion assays were performed essentially as described [18], [53], with minor modifications. *Shigella* strain M90T was a gift from Marcia Goldberg. *Shigella* strain ΔmxiC as well as pUC19-mxiC have been previously described, and were gifts from Ariel Blocker [18]. The pmxiC-RDR and pmxiC-R334D were made by standard molecular biology methods and used to transform *Shigella* strain ΔmxiC. Strains were grown on tryptic soy broth (TSB) plates containing 100 µg/mL congo red, with appropriate antibiotics. Colonies were selected and grown overnight in liquid TSB broth at 37°C and harvested by centrifugation. Pellets were resuspended in 5 mLs of fresh liquid medium. A fraction, ∼1∶25 final dilution, of the resuspended cultures was added to 50 mL TSB cultures and grown to an optical density of 1.0 (600 nm). Cultures were harvested by centrifugation, washed with warm media, and resuspended to a final OD_600_ of 5.0 in PBS+100 mg/mL Congo Red at 37°C for 10 min and 30 min. Samples were analyzed by SDS-Page using both coomassie and silver staining, as well as western blotting. Western blotting was done with an α-MxiC antibody primary, which was a gift from Ariel Blocker, and goat anti-rabbit secondary antibody (LI-COR Biosciences). Blots were developed with an Odyssey fluorescent scanner. Protein bands were identified from Mass Spectrometry of trypsin-digested bands excised from coomassie-stained gels and was performed by the Vanderbilt University Proteomics Core.

## Supporting Information

Figure S1 A Wall-eyed stereo-view of the Scc3-CopN_Δ84_ structure. 2mFo-DFc electron density, displayed at 1.5 σ is overlaid on the structure. The map was calculated in Phenix [1]. The GBR is shown in salmon and CopN is shown in green. The YopN-like domain is shown in dark green and the TyeA like domain is shown in light green.
(TIF)

Figure S2 A gatekeeper sequence alignment reveals that although sequence identity is limited, the chaperone binding regions are conserved. Representative sequences from six genera of pathogenic bacteria are shown along with the secondary structural elements observed in the CopN structure. As described in the methods, chimeric sequences were generated for the two-component gatekeepers by combining YopN and TyeA fragments. The YopN-TyeA domain boundary, the Scc3 interacting sites, and the highly conserved arginine are labeled. Strictly conserved residues are white on a red background. Residues conserved in 5 of the 7 sequences are red on a white background. Residues conserved in fewer that 5 sequences are black.
(PDF)

Figure S3 Circular Dichroism spectra of proteins used in this study confirms that all mutants encode well-folded proteins. **A.** An overlay of spectra from Scc3 and Δ24Scc3. **B.** An overlay of spectra from CopN_Δ84_ and CopN_Δ84__RDR. **C.** An overlay of spectra from MxiC_Δ74_, MxiC_Δ74__RDR, and MxiC_Δ74__R334D. All proteins show distinct minima at 208 and 222 nM, consistent with significant alpha helical content. Differences in protein concentration result in changes in the extent of minima.
(TIF)

Figure S4 CopN-Scc3 binding experiments indicate that both site 1 and site 2 are needed for complex formation. Analytical gel-filtration was used to determine the importance of the two sites of interaction in the CopN-Scc3 complex. **A.** CopN_Δ84_ binds directly to Scc3 as judged by the shift to a single, faster migrating, peak when both components are present. Both the change in retention time and the presence of both components in the eluted peak, shown in the gel to the right, indicate complex formation. **B.** Deletion of the amino terminal 24 amino acids of Scc3 (Scc3_Δ24_) disrupts CopN binding as judged the lack of co-migration on the gel-filtration column. **C.** Mutation of site 2 on CopN disrupts Scc3 binding as judged by lack of co-migration on the gel-filtration column. CopN_Δ84_RDR is mutated at three residues in site 1 (G369R, A362R, and R365D). R365 is the central arginine in site 2.
(TIF)

Figure S5 Isothermal Titration Calorimetry reveals similar translocator peptide binding characteristics for Scc3 and eh Scc3-CopN_Δ84_ complex. Heat evolved per injection is plotted in the top panel. The lower panel shows these data (solid boxes) and a best-fit model fit as a single term with non-interacting sites. The averaged Kd values, from four independent experiments, and standard deviations are shown.
(TIF)

Figure S6 The MxiC_Δ74_-R334D mutant is sufficient to disrupt type three secretion. This experiment was performed exactly as in Figure 4A, revealing that complementation of a MxiC deleted strain with MxiC_Δ74_-R334D is unable to restore wild type secretion.
(TIF)

Figure S7 Gel-filtration indicates that Scc3 is a monomer and Scc3-CopN_Δ84_ is a heterodimer. **A.** An FPLC chromatograph showing Scc3, CopN, and molecular weight standards. Scc3 and IpgC run at distinctly different retention volumes on a gel-filtration column, with Scc3 running significantly faster, consistent with dimeric IpgC and monomeric Scc3. IpgC has been previously reported to be a dimer [2], [3]. **B.** An FPLC chromatograph showing Scc3, CopN_Δ84_, Scc3-CopN_Δ84_, and molecular weight standards. **C.** A least squares fit line between Ln(MW) standards and retention volume reveals a linear relationship from 17–670 KDa. **D.** The data in panels **A.** and **B.** and the equation shown in **C.** reveal that Scc3 is a monomer, IpgC is a dimer, CopN is most reasonably interpreted as a monomer. CopN is oblong (Figure 1) and thus runs larger than expected by gel-filtration. The Scc3-CopN_Δ84_ complex is a heterodimer, composed of an oblong CopN_Δ84_ and an approximately spherical Scc3.
(TIF)

Methods S1 Supporting Methods.
(DOC)
