# Supplementary figures and images for: A Gatekeeper Chaperone Complex Directs Translocator Secretion during Type Three Secretion
Source: PLoS Pathog. 2014 Nov 6;10(11):e1004498. doi: 10.1371/journal.ppat.1004498 (PMC4222845; doi:10.1371/journal.ppat.1004498)

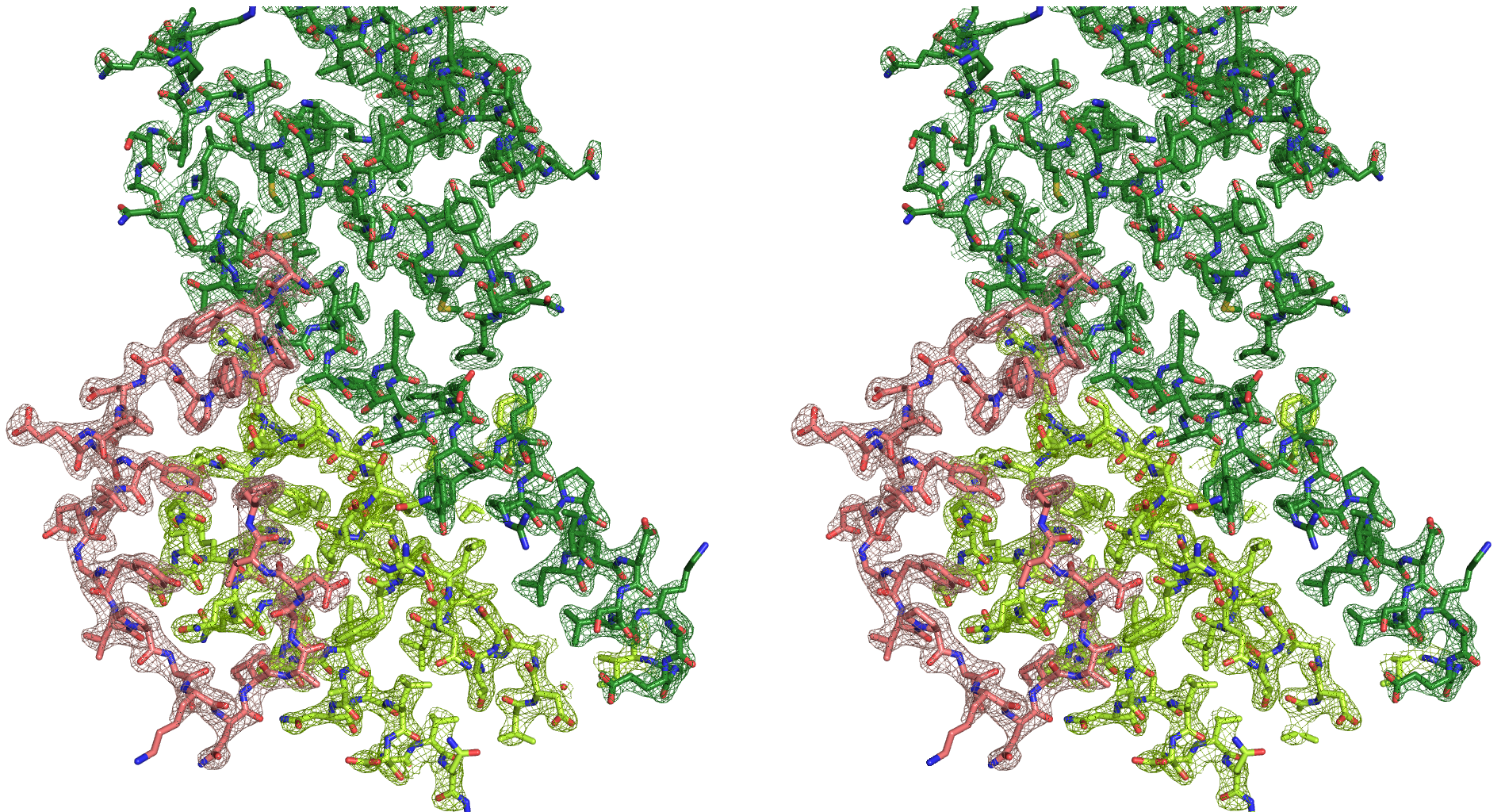

Supplement: Figure S1 — A Wall-eyed stereo-view of the Scc3-CopNΔ84 structure. 2mFo-DFc electron density, displayed at 1.5 σ is overlaid on the structure. The map was calculated in Phenix [1]. The GBR is shown in salmon and CopN is shown in green. The YopN-like domain is shown in dark green and the TyeA like domain is shown in light green. (TIF) [file ppat.1004498.s001.tif]

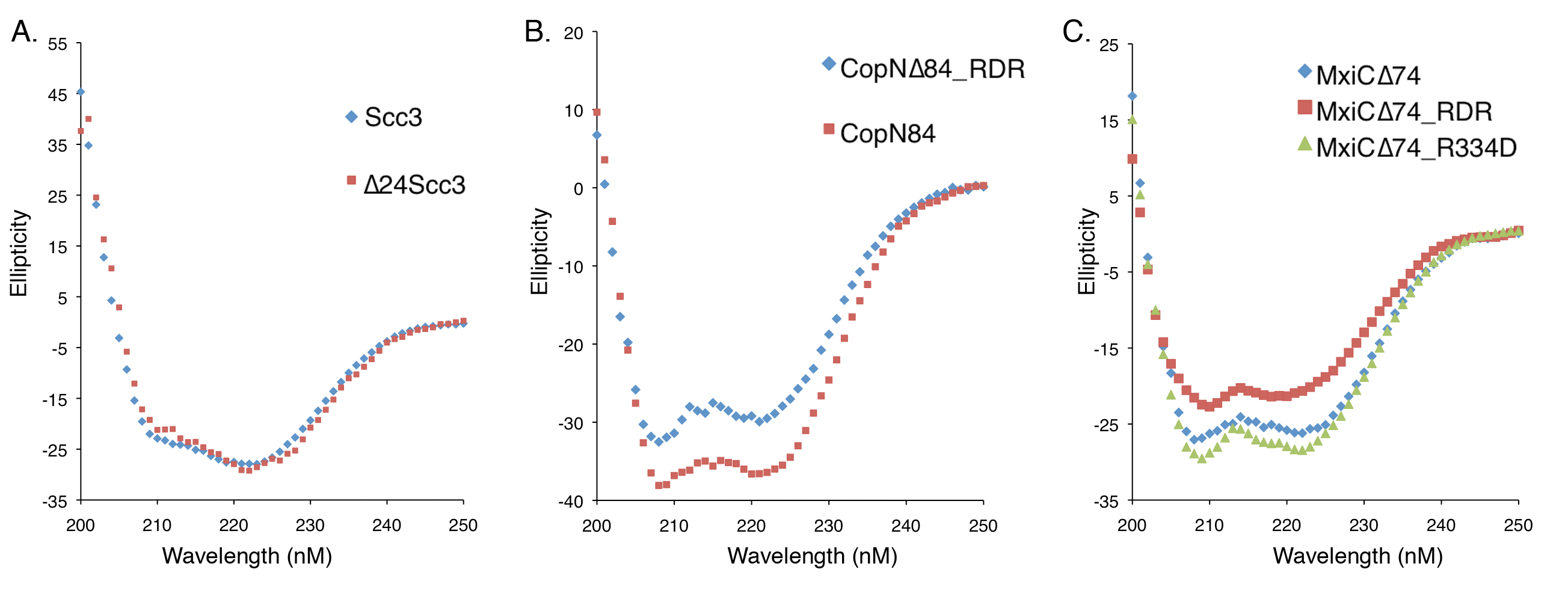

Supplement: Figure S3 — Circular Dichroism spectra of proteins used in this study confirms that all mutants encode well-folded proteins. A. An overlay of spectra from Scc3 and Δ24Scc3. B. An overlay of spectra from CopNΔ84 and CopNΔ84_RDR. C. An overlay of spectra from MxiCΔ74, MxiCΔ74_RDR, and MxiCΔ74_R334D. All proteins show distinct minima at 208 and 222 nM, consistent with significant alpha helical content. Differences in protein concentration result in changes in the extent of minima. (TIF) [file ppat.1004498.s003.tif]

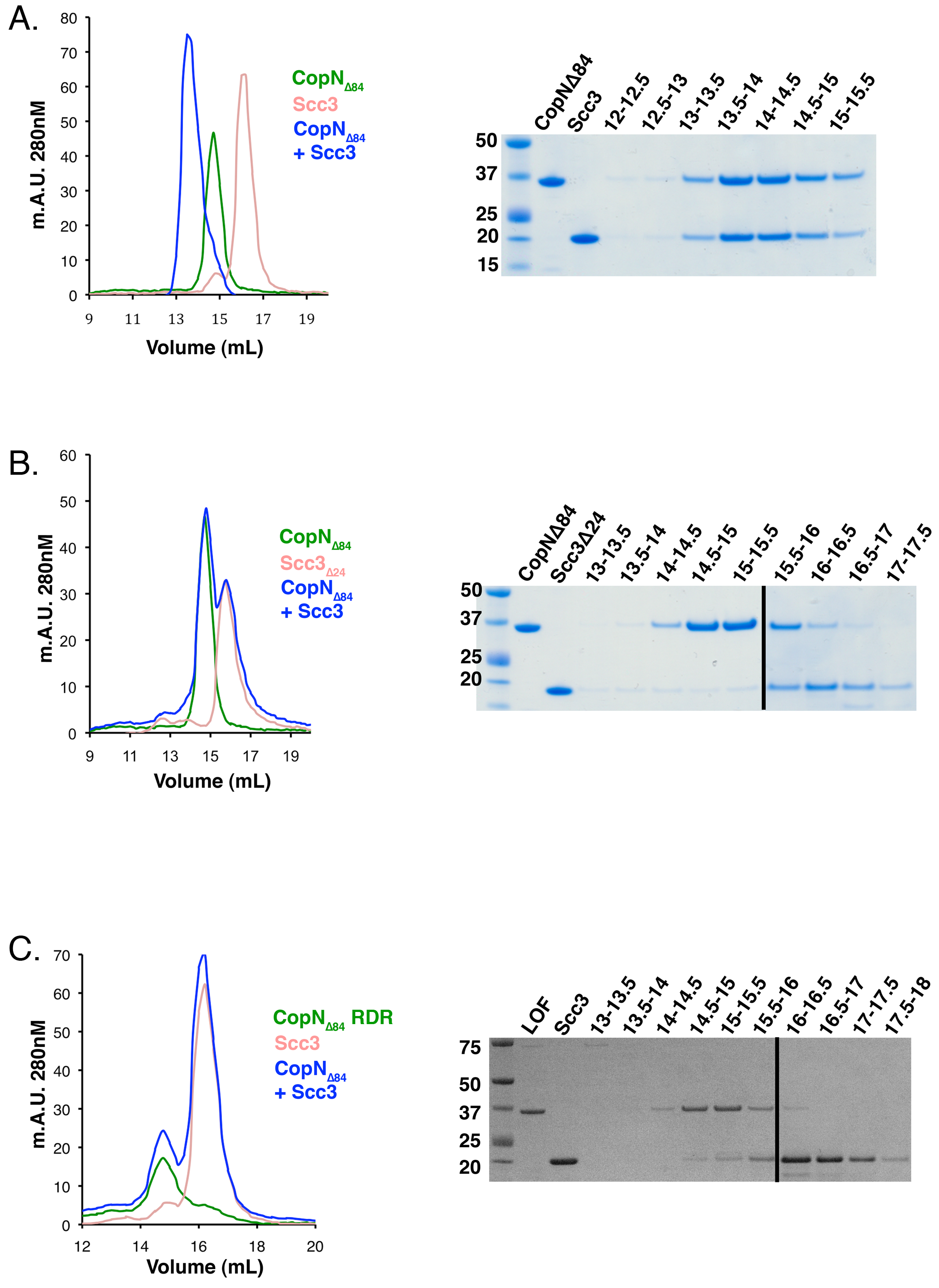

Supplement: Figure S4 — CopN-Scc3 binding experiments indicate that both site 1 and site 2 are needed for complex formation. Analytical gel-filtration was used to determine the importance of the two sites of interaction in the CopN-Scc3 complex. A. CopNΔ84 binds directly to Scc3 as judged by the shift to a single, faster migrating, peak when both components are present. Both the change in retention time and the presence of both components in the eluted peak, shown in the gel to the right, indicate complex formation. B. Deletion of the amino terminal 24 amino acids of Scc3 (Scc3Δ24) disrupts CopN binding as judged the lack of co-migration on the gel-filtration column. C. Mutation of site 2 on CopN disrupts Scc3 binding as judged by lack of co-migration on the gel-filtration column. CopNΔ84RDR is mutated at three residues in site 1 (G369R, A362R, and R365D). R365 is the central arginine in site 2. (TIF) [file ppat.1004498.s004.tif]

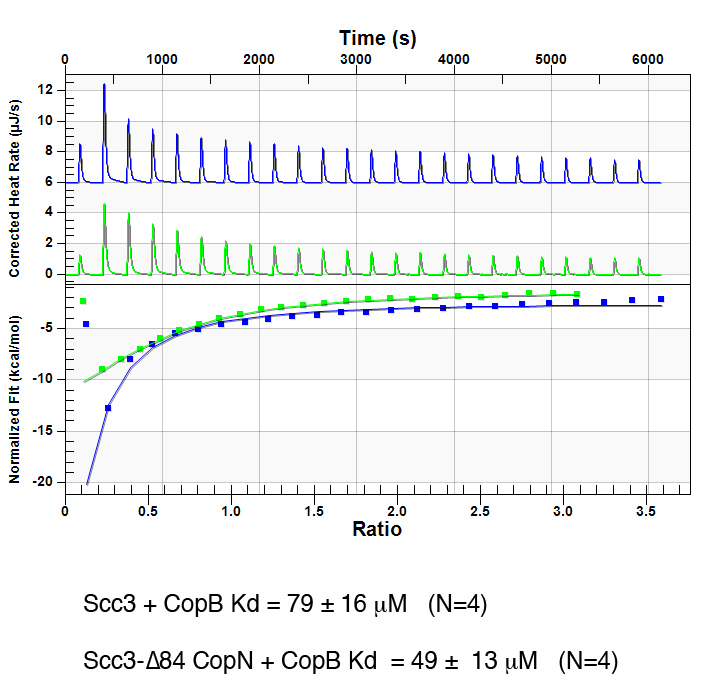

Supplement: Figure S5 — Isothermal Titration Calorimetry reveals similar translocator peptide binding characteristics for Scc3 and eh Scc3-CopNΔ84 complex. Heat evolved per injection is plotted in the top panel. The lower panel shows these data (solid boxes) and a best-fit model fit as a single term with non-interacting sites. The averaged Kd values, from four independent experiments, and standard deviations are shown. (TIF) [file ppat.1004498.s005.tif]

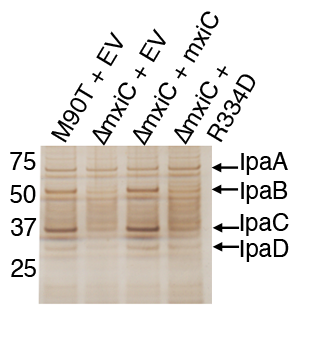

Supplement: Figure S6 — The MxiCΔ74-R334D mutant is sufficient to disrupt type three secretion. This experiment was performed exactly as in Figure 4A, revealing that complementation of a MxiC deleted strain with MxiCΔ74-R334D is unable to restore wild type secretion. (TIF) [file ppat.1004498.s006.tif]

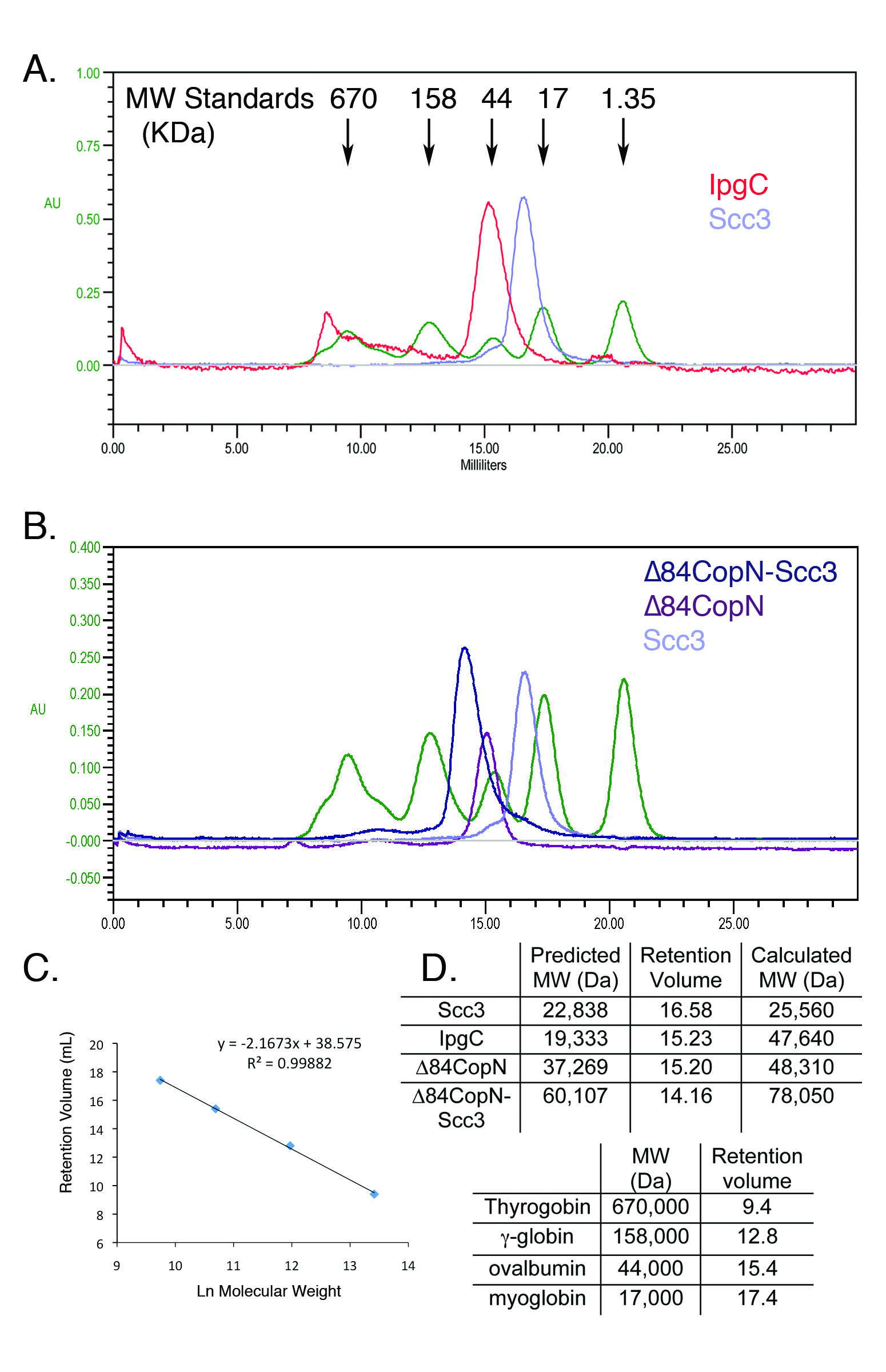

Supplement: Figure S7 — Gel-filtration indicates that Scc3 is a monomer and Scc3-CopNΔ84 is a heterodimer. A. An FPLC chromatograph showing Scc3, CopN, and molecular weight standards. Scc3 and IpgC run at distinctly different retention volumes on a gel-filtration column, with Scc3 running significantly faster, consistent with dimeric IpgC and monomeric Scc3. IpgC has been previously reported to be a dimer [2], [3]. B. An FPLC chromatograph showing Scc3, CopNΔ84, Scc3-CopNΔ84, and molecular weight standards. C. A least squares fit line between Ln(MW) standards and retention volume reveals a linear relationship from 17–670 KDa. D. The data in panels A. and B. and the equation shown in C. reveal that Scc3 is a monomer, IpgC is a dimer, CopN is most reasonably interpreted as a monomer. CopN is oblong (Figure 1) and thus runs larger than expected by gel-filtration. The Scc3-CopNΔ84 complex is a heterodimer, composed of an oblong CopNΔ84 and an approximately spherical Scc3. (TIF) [file ppat.1004498.s007.tif]
