# Supplementary material for: A Gatekeeper Chaperone Complex Directs Translocator Secretion during Type Three Secretion
Source: PLoS Pathog. 2014 Nov 6;10(11):e1004498. doi: 10.1371/journal.ppat.1004498 (PMC4222845; doi:10.1371/journal.ppat.1004498)
Supplement: Figure S2 — A gatekeeper sequence alignment reveals that although sequence identity is limited, the chaperone binding regions are conserved. Representative sequences from six genera of pathogenic bacteria are shown along with the secondary structural elements observed in the CopN structure. As described in the methods, chimeric sequences were generated for the two-component gatekeepers by combining YopN and TyeA fragments. The YopN-TyeA domain boundary, the Scc3 interacting sites, and the highly conserved arginine are labeled. Strictly conserved residues are white on a red background. Residues conserved in 5 of the 7 sequences are red on a white background. Residues conserved in fewer that 5 sequences are black. (PDF) [file ppat.1004498.s002.pdf]

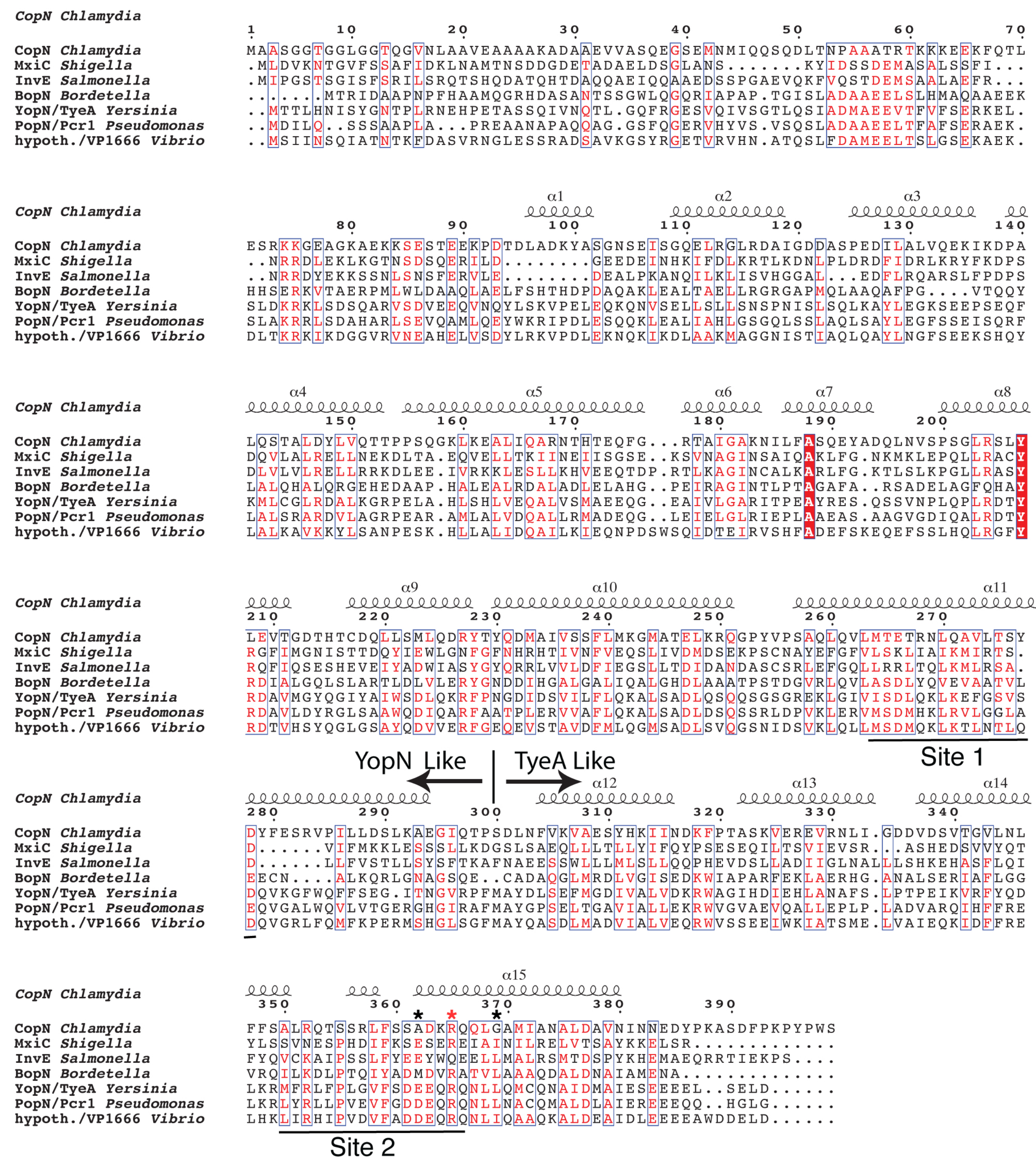
CopN Chlamydia
CopN Chlamydia MAASGGTGGLGGTQGVNLAAVEAAAAKADAAEVVASQEGSEMNMIQQSQDLTNPAAATRTKKKEEKFQTL
MxiC Shigella .MLDVKNTGVFSSAFIDKLNAMTNSDDGDETADAELDSGLANS.......KYIDSSDEMASALSSFI...
InvE Salmonella .MIPGSTSGISFSRILSRQTSHQDATQHTDAQQAEIQQAAEDSSPGAEVQKFVQSTDEMSAALAEFR...
BopN Bordetella ......MTRIDAAPNPFHAAMQGRHDASANTSSGWLQGQRIAPAP.TGISLADAAEELSLHMAQAAEEK
YopN/TyeA Yersinia ..MTTLHNISYGNTPLRNEHPETASSQIVNQTL.GQFRGESVQIVSGTLQSIADMAEEVTFVFSERKEL.
PopN/Pcr1 Pseudomonas ..MDILQ..SSSAAPLA...PREAANAPAQQAG.GSFQGERVHYVS.VSQSLADAAEELTFAFSERAEK.
hypoth./VP1666 Vibrio ..MSIINSQIATNTKFDASVRNGLESSRADSAVKGSYRGETVRVHN.ATQSLFDAMEELTSLGSEKAEK.
CopN Chlamydia
CopN Chlamydia ESRKKGEAGKAEKKSESTEEKPDTDLADKYASGNSEISGQELRGLRDAIGDDASPEDILALVQEKIKDPA
MxiC Shigella ..NRRDLEKLKGTNSDSQERILD........GEEDEINHKIFDLKRTLKDNLPLDRDFIDRLKRYFKDPS
InvE Salmonella ..NRRDYEKKSSNLSNSFERVLE........DEALPKANQILKLISVHGGAL...EDFLRQARSLFPDPS
BopN Bordetella HHSERKVTAERPMLWLDAAQLAELFSHTHDPDAQAKLEALTAELLRGRGAPMQLAAQAFPG....VTQQY
YopN/TyeA Yersinia SLDKRKLSDSQARVSDVEEQVNQYLSKVPELEQKQNVSELLSLLSNSPNISLSQLKAYLEGKSEEPSEQF
PopN/Pcr1 Pseudomonas SLAKRRLSDAHARLSEVQAMLQEYWKRIPDLESQQKLEALIAHLGSGQLSSLAQLSAYLEGFSSEISQRF
hypoth./VP1666 Vibrio DLTKRKIKDGGVRVNEAHELVSDYLRKVPDLEKNQKIKDLAAKMAGGNISTIAQLQAYLNGFSEEKSHQY
CopN Chlamydia
CopN Chlamydia LQSTALDYLVQTTPPSQGKLKEALIQARNTHTEQFG...RTAIGAKNILFASQEYADQLNVSPSGLRSLY
MxiC Shigella DQVLALRELLNEKDLTA.EQVELLTKIINEIISGSE..KSVNAGINSAIQAKLFG.NKMKLEPQLLRACY
InvE Salmonella DLVLVLRELLRRKDLEE.IVRKKLESLLKHVEEQTDP.RTLKAGINCALKARLFG.KTLSLKPGLLRASY
BopN Bordetella LALQHALQRGEHEDAAP.HALEALRDALADLELAHG..PEIRAGINTLPTAGAFA..RSADELAGFQHAY
YopN/TyeA Yersinia KMLCGLRDALKGRPELA.HLSHLVEQALVSMAEEQG..EAIVLGARITPEAYRES.QSSVNPLQPLRDTY
PopN/Pcr1 Pseudomonas LALSRARDVLAGRPEAR.AMLALVDQALLRMADEQG..LEIELGLRIEPLAAEAS.AAGVGDIQALRDTY
hypoth./VP1666 Vibrio LALKAVKKYLSANPESK.HLLALIDQAILKIEQNPDSWSQIDTEIRVSHFADEFSKEQEFSSLHQLRGFY
CopN Chlamydia
CopN Chlamydia LEVTGDTHTCDQLLSMLQDRYTYQDMAIVSSFLMKGMATELKRQGPYVPSAQLQVLMTETRNLQAVLTSY
MxiC Shigella RGFIMGNISTTDQYIEWLGNFGFNHRHTIVNFVEQSLIVDMDSEKPSCNAYEFGFVLSKLIAIKMIRTS.
InvE Salmonella RQFIQSESHEVEIYADWIASYGYQRRLVVLDFIEGSLLTDIDANDASCSRLEFGQLLRRLTQLKMLRSA.
BopN Bordetella RDIALGQLSLARTLDLVLERYGNDDIHGALGALIQALGHDLAAATPSTDGVRLQVLASDLYQVEVAATVL
YopN/TyeA Yersinia RDAVMGYQGIYAIWSDLQKRFPNGDIDSVILFLQKALSADLQSQQSGSGREKLGIVISDLQKLKEFGSVS
PopN/Pcr1 Pseudomonas RDAVLDYRGLSAAWQDIQARFAATPLERVVAFLQKALSADLDSQSSRLDPVKLERVMSDMHKLRVLGGLA
hypoth./VP1666 Vibrio RDTVHSYQGLGSAYQDVVERFGEQEVSTAVDFMLQGMSADLSVQGSNIDSVKLQLLMSDMQKLKTLNTLQ
Site 1
YopN Like
TyeA Like
CopN Chlamydia
CopN Chlamydia DYFESRVPILLDSLKAEGIQTPSDLNFVKVAESYHKIINDKFPTASKVEREVRNLI.GDDVDSVTGVLNL
MxiC Shigella D......VIFMKKLESSSLLKDGSLSAEQLLLTLLYIFQYPSESEQILTSVIEVSR...ASHEDSVVYQT
InvE Salmonella D.......LLFVSTLLSYSFTKAFNAEESSWLLLMLSLLQQPHEVDSLLADIIGLNALLSHKEHASFLQI
BopN Bordetella EECN....ALKQRLGNAGSQE..CADAQGLMRDLVGISEDKWIAPARFEKLAERHG.ANALSERIAFLGG
YopN/TyeA Yersinia DQVKGFWQFFSEG.ITNGVRPFMAYDLSEFMGDIVALVDKRWAGIHDIEHLANAFS.LPTPEIKVRFYQD
PopN/Pcr1 Pseudomonas EQVGALWQVLVTGERGHGIRAFMAYGPSELTGAVIALLEKRWVGVAEVQALLEPLP.LADVARQIHFFRE
hypoth./VP1666 Vibrio DQVGRLFQMFKPERMSHGLSGFMAYQASDLMADVIALVEQRWVSSEEIWKIATSME.LVAIEQKIDFFRE
CopN Chlamydia
CopN Chlamydia FFSALRQTSSRLFSSADKRQQLGAMIANALDAVNINNEDYPKASDFPKPYPWS
MxiC Shigella YLSSVNESPHDIFKSESEREIAINILRELVTSAYKKELSR.............
InvE Salmonella FYQVCKAIPSSLFYEEYWQEELLMALRSMTDSPYKHEMAEQRRTIEKPS....
BopN Bordetella VRQILKDLPTQIYADMDVRATVLAAQDALDNAIAMENA..............
YopN/TyeA Yersinia LKRMFRLFPLGVFSDEEQRQNLLQMCQNAIDMAIESEEEL..SELD......
PopN/Pcr1 Pseudomonas LKRLYRLLPVEVFGDDEQRQNLLNACQMALDLAIEREEEQ..HGLG......
hypoth./VP1666 Vibrio LHKLIRHIPVDVFADDEQRQNLIQAAQKALDEAIDLEEEEAWDDELD......
Site 2
