# Supplementary material for: A Gatekeeper Chaperone Complex Directs Translocator Secretion during Type Three Secretion
Source: PLoS Pathog. 2014 Nov 6;10(11):e1004498. doi: 10.1371/journal.ppat.1004498 (PMC4222845; doi:10.1371/journal.ppat.1004498)
Supplement: Methods S1 — Supporting Methods. (DOC) [file ppat.1004498.s008.doc]

**Method S1:**

**Circular Dichroism (CD):** To assure that mutant proteins were properly folded, CD spectra were measured for all proteins used in this study. Spectra were recorded on a Jasco J-810 spectropolarimeter at 20 ˚C in 10 mM NaPhosphate, 150 mM NaCl, pH 7.5. Proteins were purified as described in the main methods section and diluted to appropriate concentrations (~0.1 mg/mL) so as to maintain the photomultiplier tube voltage below 500 V.

**Isothermal Titration Calorimetry (ITC):** Experiments were conducted on a on a Nano ITC instrument from TA instruments at 20 °C. 25 injections of 2 μL each into a 300 μL sample with a 120 s interval between injections were measured. CopB derived peptide (LETPELPKPGV, 95% pure) from GenScript was used at 2 mM in the injection syringe. The sample cell contained either Scc3 at 215 μM or Scc3-CopN_∆84_ complex at 215 μM in 10mM Tris, 150 mM NaCl, 0.5 mM TCEP, pH 7.5. Titration data were analyzed with software from TA instruments. Four replicate experiments were performed and average dissociation constants and standard deviations are reported.

**Supporting References.**

1. Adams PD, Grosse-Kunstleve RW, Hung LW, Ioerger TR, McCoy AJ, et al. (2002) PHENIX: building new software for automated crystallographic structure determination. Acta Crystallogr D Biol Crystallogr 58: 1948-1954.

2. Adam P, Patil M, Dickenson N, Choudhari S, Barta M, et al. (2012) Binding affects the tertiary and quaternary structures of the Shigella translocator protein IpaB and its chaperone IpgC. Biochemistry 51: 4062-4071.

3. Harrington AT, Hearn PD, Picking WL, Barker JR, Wessel A, et al. (2003) Structural characterization of the N terminus of IpaC from Shigella flexneri. Infect Immun 71: 1255-1264.
